# Turning threats into opportunities: how to implement and advance quality TB services for people with HIV during the COVID‐19 pandemic and beyond

**DOI:** 10.1002/jia2.25696

**Published:** 2021-03-31

**Authors:** Teri Roberts, Suvanand Sahu, James Malar, Timur Abdullaev, Wim Vandevelde, Yogan G Pillay, Paula I Fujiwara, Alasdair Reid, Shannon Hader, Satvinder Singh, Adeeba Kamarulzaman, Sevim Ahmedov

**Affiliations:** ^1^ International AIDS Society Geneva Switzerland; ^2^ Stop TB Partnership Geneva Switzerland; ^3^ TBPeople Tashkent Uzbekistan; ^4^ GNP+ Johannesburg South Africa; ^5^ Clinton Health Access Initiative Pretoria South Africa; ^6^ International Union Against Tuberculosis and Lung Disease Paris France; ^7^ UNAIDS Geneva Switzerland; ^8^ World Health Organization Geneva Switzerland; ^9^ University of Malaya Kuala Lumpur Malaysia; ^10^ United States Agency for International Development Washington DC USA

**Keywords:** tuberculosis, HIV, COVID‐19, differentiated service delivery, innovation, integration, stigma, people affected by TB

## Abstract

**Introduction:**

Until COVID‐19, tuberculosis (TB) was the leading infectious disease killer globally, disproportionally affecting people with HIV. The COVID‐19 pandemic is threatening the gains made in the fight against both diseases.

**Discussion:**

Although crucial guidance has been released on how to *maintain* TB and HIV services during the pandemic, it is acknowledged that what was considered normal service pre‐pandemic needs to *improve* to ensure that we rebuild person‐centred, inclusive and quality healthcare services. The threat that the pandemic may reverse gains in the response to TB and HIV may be turned into an opportunity by pivoting to using proven differentiated service delivery approaches and innovative technologies that can be used to maintain care during the pandemic and accelerate improved service delivery in the long term. Models of care should be convenient, supportive and sufficiently differentiated to avoid burdensome clinic visits for medication pick‐ups or directly observed treatments. Additionally, the pandemic has highlighted the chronic and short‐sighted lack of investment in health systems and the need to prioritize research and development to close the gaps in TB diagnosis, treatment and prevention, especially for children and people with HIV. Most importantly, TB‐affected communities and civil society must be supported to lead the planning, implementation and monitoring of TB and HIV services, especially in the time of COVID‐19 where services have been disrupted, and to report on legal, policy and gender‐related barriers to access experienced by affected people. This will help to ensure that TB services are held accountable by affected communities for delivering equitable access to quality, affordable and non‐discriminatory services during and beyond the pandemic.

**Conclusions:**

Successfully reaching the related targets of ending TB and AIDS as public health threats by 2030 requires rebuilding of stronger, more inclusive health systems by advancing equitable access to quality TB services, including for people with HIV, both during and after the COVID‐19 pandemic. Moreover, services must be rights‐based, community‐led and community‐based, to ensure that no one is left behind.

## 1 INTRODUCTION

Until COVID‐19, tuberculosis (TB) was the leading infectious disease killer globally, including among people with HIV [1], who are disproportionally affected by TB [2, 3]. In 2019, at least 1.2 million people died from TB, of which 12% were children and 208,000 had HIV, reflecting a third of AIDS deaths [1]. The Political Declaration on the Fight against Tuberculosis resulting from the United Nations High Level Meeting (UNHLM) on TB in September 2018 [4] committed world leaders to a number of targets by 2022, including prioritizing high‐risk populations, which include people with HIV, and providing TB preventive treatment (TPT) to six million people with HIV [5], with the latter insufficiently ambitious and warranting review. Thus, successfully reaching the targets of ending TB and ending AIDS as a public health threat by 2030 are clearly intertwined.

Important progress has been made in the global response to TB and HIV, with an average decline in the TB cumulative incidence rate of 2.3% between 2018 and 2019 [1] and 25.4 million of the 38 million people with HIV on antiretroviral treatment (ART), along with a 23% decline in new HIV infections since 2010 [3]. However, since the beginning of 2020, the world has seen the devastating effects of the COVID‐19 pandemic threatening to undermine and undo a decade’s worth of progress due to lockdowns, limited access to health facilities and stigma. A modelling study performed in 2020 projected that a three‐month global lockdown followed by a ten‐month restoration period could lead to an additional 6.3 million cases of TB between 2020 and 2025 and an increase in global TB incidence and deaths in 2021 to levels last seen in 2013 and 2016, respectively – a setback of at least five to eight years [6]. The Global Fund have been documenting how COVID‐19 has been affecting the global response to HIV, TB and malaria [7], with surveys revealing disruptions to both TB and HIV programmes in three‐quarters of countries and around 15% reporting high to very high disruption.

Due to the need to mitigate the impact of COVID‐19 on TB services, including for people with HIV [8], a group of ten TB civil society and affected community organizations and networks undertook a survey from the 26 May to the 2 July 2020, which detailed TB‐affected community and civil society perspectives and priorities pertaining to the impact of the pandemic. The concerning results show a devastating impact and a subsequent call to action included the need for the development of a TB recovery plan and a comprehensive, rights‐based response to TB and COVID‐19 that meaningfully engages TB‐affected communities, addresses legal and social barriers to accessing services and scales‐up social protection systems [9]; and others have published guidance on how to maintain TB and HIV services during the pandemic [10].

Specifically for people with HIV, those who are immunocompromised may be at higher risk of a worse outcome from COVID‐19 and a study from South Africa showed that increased COVID‐19 mortality was associated with HIV and previous and current TB, among other variables [11]. This is concerning given that there was a halving of TB testing during the lockdown as well as a decline in the collection of TB and HIV medications [12].

While the priority for countries is to safely maintain services during the pandemic, it is necessary to also build stronger, more inclusive systems for health that may achieve a TB‐free world [13]. Here we discuss how to improve TB services in the wake of the current COVID‐19 pandemic through using current and future innovations in models of care and tools, implemented within a human rights‐based response.

## 2 DISCUSSION

### 2.1 The need to shift to better and more integrated models of care

Even within the constraints of physical distancing requirements imposed by the public health response to COVID‐19, much progress can be made in improving TB services, with the provision of counselling and peer support, through improved models of care and ensuring that new and approved tools are made accessible at scale as soon as possible. While HIV programmes have been implementing differentiated service delivery (DSD) approaches in many settings [14], during the pandemic, many countries have accelerated transition to these innovative and proven alternative models (Table 1), including integrating HIV, TB and COVID‐19 services, to meet the most immediate health needs of their populations while ensuring physical distancing to limit the spread of SARS‐CoV‐2 [12, 15], as they reduce the need for people to go to health facilities where the risk of exposure to COVID‐19 is higher, while ensuring that TB services remain safe and functional to avoid diagnostic delays and treatment interruption [16]. Moreover, as both diseases can present similarly with respiratory symptoms and fever, have airborne transmission and require similar prevention efforts, a joint and integrated approach in disease diagnosis, contact tracing and infection prevention and control (IPC) is recommended (Table 1) [17]. Considering that hospitals are designed more for bloodborne than airborne infection control, subsequent improvements made in droplet and aerosol IPC following COVID‐19 should be leveraged for TB control in endemic settings to prevent infection of both healthcare workers and people with TB.

**Table 1:** Recommendations for implementing and advancing quality TB services for people with HIV during the COVID‐19 pandemic and beyond

Recommendation	Example
Better and more integrated models of care	
Implement and expand access to DSD [14, 15]	Offer TB screening and TPT Offer multi‐month dispensing of TB treatment and TPT Integrate community service delivery Provide community‐led contact tracing Offer adherence support clubs
Improve IPC for airborne pathogens to benefit COVID‐19 and TB control [17]	Provide PPE to healthcare workers Address structural problems with ventilation in health facilities Use ultraviolet light for disinfection more widely
Scale‐up the use of TPT [18]	Offer preferably 1HP^a^ or 3HP^b^ TPT to all contacts of people with active pulmonary TB and people of all ages with HIV for whom active TB disease has been ruled out
Scale‐up use of the TB LAM screening test [19]	Offer the TB LAM test to all people with HIV with TB symptoms to help increase diagnosis at facilities
Scale‐up use of molecular testing for TB [20]	Ensure that molecular testing to measure *M. tuberculosis* (MTB) and rifampicin (RIF) resistance is comprehensively available
Integrate screening for HIV, TB and COVID‐19 [21, 22]	Use shared technologies, such as Cepheid’s GeneXpert system that can diagnose TB and COVID‐19 and measure HIV viral load simultaneously Offer testing for HIV, TB and COVID‐19 for symptomatic individuals presenting for SARS‐CoV‐2 testing in high TB and HIV burden settings Increase testing capacity through procurement of additional instrumentation and strengthening laboratory capacity Apply HIV and TB expertise in contact tracing to COVID‐19 Scale‐up use of the digital health toolkit for TB
Ensure a broader commitment to the empowerment of people affected by TB and other diseases [23, 24]	Retain successful models post‐pandemic within a human rights‐based response
New TB tools to improve care during and after COVID‐19	
Switch to all oral regimens for drug‐resistant TB [25]	Discontinue injectable treatments by using bedaquiline‐based all oral regimens for drug‐resistant TB
Limit in‐person contact with the healthcare system while retaining quality, person‐centred care	Replace directly observed treatment with other forms of supported care, including self‐administered therapy with psychosocial support and, potentially, digital monitoring if feasible and acceptable to people, employing multimodal forms of support and multi‐month dispensing (as is already the case with nearly every other disease) Use the smart‐phone‐based application called “ONE IMPACT” [26] (available in 11 languages) to support community‐based monitoring and containing information about TB, where to access TB services, how to connect to other people with TB or support groups, and how to get involved and report problems on accessing quality TB services Use tele‐radiology and interpretation of chest X‐rays using artificial intelligence Use tele‐health solutions such as mobile applications for case detection Use connectivity solutions to upload anonymized results directly to a cloud‐based server to speed up turn‐around time of results and linkage to care for individuals and to support disease surveillance, programme quality control and supply management at programmatic level
Continue development of new and improved tools	Ensure sufficient resources are available to continue the development and sustainable implementation of new and improved diagnostics, treatments and vaccines
Human rights and stigma	
Implement and sustain commitments from the United Nations High Level Meeting on TB (September 2018)	These include access to psychosocial, nutritional and adherence support and the elimination of stigma and discrimination
Decrease legal, policy and gender‐related barriers to accessing TB care	Support TB‐affected communities and civil society to help ensure adequate and human rights‐based social protection and health systems

DSD, differentiated service delivery; IPC, infection prevention and control; PPE, personal protective equipment; TB, tuberculosis; TPT, TB preventative therapy.

^a^ 1HP: 4 weeks of daily isoniazid (H) and rifapentine (P).

^b^ 3HP: 12 weeks of isoniazid (H) and rifapentine (P), once weekly.

TPT can also be provided in a DSD model, preferably as an affordable, once‐a‐day, fixed‐dose combination (FDC) [18], which may facilitate the urgent need for scale‐up, particularly important in the time of COVID‐19 when people are presenting less for diagnosis of active TB. The WHO guidelines include multiple TPT options, including the shorter, better tolerated, rifamycin‐based TPT regimens, including the “game‐changing” options 1HP (four weeks of daily isoniazid (H) and rifapentine (P)) and 3HP (12 weeks of H and P, once weekly, now available as a FDC), instead of six months of daily isoniazid [18, 27]. Importantly, these are safe with dolutegravir‐ and efavirenz‐based ART and do not require dose adjustment [28].

For TB screening, TB lipoarabinomannan (TB LAM) is present in the urine and may be used to rule‐in active TB. The Alere Determine TB LAM Ag test (Abbott) is recommended by the WHO for use in people with HIV with TB symptoms [19] to rule‐in TB, and also requires urgent scale‐up, including in DSD models (Table 1). As the test is inexpensive and easy to perform, whether at the hospital, clinic or community‐based settings, this can be leveraged to massively increase screening for TB in people with HIV in a feasible way, thereby facilitating the quicker diagnosis of people most likely to have a worse TB prognosis and who urgently require linkage to care and treatment [29]. This is particularly advantageous in the time of COVID‐19 when people may be reluctant to seek a TB diagnosis at the facility level but where LAM screening can easily be done in decentralized settings. Although the pooled sensitivity of 42% in people with HIV with TB symptoms is low, specificity is high at 91% [30], and sensitivity improves incrementally with more pronounced immunosuppression. This means that the test plugs a crucial gap in helping to diagnosis people with HIV who have TB but are negative by sputum smear and even Xpert MTB/RIF (Cepheid) [31]. Additionally, access to Xpert MTB/RIF needs urgent scale‐up as, of the 37 countries included in the Step Up for TB report, only one in seven people have access to a rapid molecular test [20].

Lessons learnt to improve active TB case finding performed through ZAMSTAR [32] and the HPTN 071 (PopART) trial [33] have now been extended to COVID‐19 through the ongoing TREATS project clinical trial [21], an exemplary example of integrated community‐based screening for TB, HIV and COVID‐19 (Table 1). With regard to using shared technologies for diagnosis and monitoring of HIV, TB and COVID‐19, while seemingly streamlined, it should be noted that there is often the need for multiple and different clinical samples to be obtained for this menu of testing, making it less straightforward for healthcare providers and people alike. Moreover, worthwhile additions would be to fast track access to affordable point‐of‐care tests for TB, to expand integrated testing to contacts of people diagnosed with TB or COVID‐19, and to increase access to hand‐held x‐ray equipment.

### 2.2 The need to introduce new tools to improve TB care in the context of the COVID‐19 pandemic and beyond

Tests that are as simple to use as the current Determine TB LAM Ag test but that are more sensitive at higher CD4 T cell counts in people with HIV are in the near pipeline. This will help to increase the number of people with HIV being successfully screened for TB in decentralized settings during the COVID‐19 pandemic and beyond. The first of these, the Fujifilm SILVAMP TB LAM (FujiLAM) assay, is already undergoing clinical trials for regulatory approval and WHO endorsement. To improve the sensitivity even further, additional work on other next‐generation TB LAM tests is underway.

Shorter, less complicated and more tolerable TB treatment regimens will also greatly aid successful care during the COVID‐19 pandemic as it will facilitate less interaction with health facilities through reduced need for direct adherence support, toxicity monitoring via laboratory testing, and length of interaction with healthcare facilities. This is especially true for the treatment of drug‐resistant TB (DR‐TB). The WHO has already recommended fully oral, bedaquiline‐based regimens for the majority of people with DR‐TB, which are compatible with current ART regimens, thus dropping the painful and toxic injectable treatments [25]. Furthermore, many trials are underway to support the next guideline review on shorter, all‐oral regimens of six to twelve months in duration, including the new and much anticipated “BPaL” regimen (containing bedaquiline, high‐dose linezolid and pretomanid) for extensively drug‐resistant TB or multidrug‐resistant TB that does not respond to treatment. The 31/A5349 [34] and SHINE [35] studies have also shown that 4‐month regimens for drug‐sensitive TB are possible, including for people with HIV. The addition of three new drugs (bedaquiline, delamanid and pretomanid) puts a dent in the otherwise enormous paucity of new and improved medications for TB and DR‐TB, but much effort will be needed to ensure global implementation at scale to reach all in need. Additionally, the ideal “one month (or less) treatment regimen for all types of TB, which works for everyone, everywhere”, which is also fully compatible with all WHO‐recommended ART regimens, remains an unmet objective [36].

The COVID‐19 pandemic has, by necessity, fast‐tracked a number of approaches, some aided by digital tools, to limit in‐person contact with the healthcare system. These should be retained longer‐term where evidence shows that they sustain and improve service quality and retention in care, which is currently very poor, especially for those with DR‐TB, with HIV, or especially for children [37, 38]. On the other hand, caution must be taken that sufficient support be provided when replacing in person services, such as directly observed treatment (DOT), with remote counselling to ensure successful treatment (Table 1).

One hope for the future is in the availability of vaccines for TB and HIV, as has been recently demonstrated for COVID‐19. No vaccine has yet been launched for HIV, and the existing vaccine for TB, Bacillus Calmette–Guérin (BCG), is sub‐optimal and does not interrupt transmission. A number of novel TB vaccine candidates are in the pipeline [39], such as DAR901 [40] and M72/AS01E [41], the latter of which provides 54% protection against pulmonary TB disease in infected adults, and is the first moderately effective vaccine against TB disease in adults to be developed in almost 100 years. Given the slow pace of vaccine development for TB and HIV, it is also hoped that the successes, in such a short space of time, for the development of COVID‐19 vaccines prove that with sufficient political will and resources, vaccine development for other infectious diseases can be much more efficient.

### 2.3 The need to promote human rights and reduce stigma

The UNHLM on TB recognizes that, for many people affected by TB, the existence of legal, social and economic barriers inhibits their access to the TB prevention, diagnosis, treatment, care and support they need. The UNHLM on TB contains commitments to eliminate stigma and discrimination, to provide access to psychosocial, nutritional and adherence support – that does not rely on direct observation of the person taking their treatment but on treatment and disease literacy and giving the person agency over their own treatment – to overcome legal barriers to access, promote TB responses that are guided by principles of human rights and gender equality, and reach and meaningfully engage those most marginalized and vulnerable, TB key populations (Table 1) [4]. During the time of COVID‐19 and beyond, where additional economic hardships have affected already vulnerable people, protections must be put in place to avoid the enormous and often catastrophic economic and social impacts of TB [5].

Despite the fact that person‐centred psycho‐emotional and socio‐economic interventions improve TB treatment outcomes [42], psychosocial, nutritional and adherence support are generally lacking in TB care, including for the treatment of comorbid depression and substance use [43]. It is therefore recommended that more focus should be placed on, and resources allocated to, these under‐researched and under‐addressed issues. For example in Peru, despite stigma and fear, people benefitted from psychosocial support following disclosure and recommended improving support further through education of communities on TB and improved nutritional support and vocational activities for themselves [44]. Psychosocial support improves retention in care for people with DR‐TB [45] and provides crucial rehabilitation after treatment completion [46].

To this end, TB‐affected communities and civil society have been supported [4] to conduct assessments on legal, policy and gender‐related barriers to access that are experienced by people with TB [47] in 15 countries so far [48]. In addition, costed, TB communities, rights and gender (CRG) Action Plans have been developed and integrated into several countries’ National Strategic Plans. This process of operationalizing a rights‐based response has been advanced by TB‐affected communities and civil society themselves, including through the *Declaration of the Rights of People Affected by TB* [23], *Right to Breathe* [23], *Activating A Human Rights TB Response* [23], *A Deadly Divide* [49], as well as through the development of various CRG Investment Packages [48], including a TB Stigma Assessment [47].

During the COVID‐19 pandemic, inequalities and human rights barriers have been exacerbating the marginalization and vulnerability experienced by millions. According to TB‐affected communities, social protection systems remain inaccessible, as do the health services they need [9], impacting COVID‐19 and other health services. Community‐led monitoring initiatives of the availability, accessibility, acceptability and quality of services [26] have therefore never been more important in ensuring people with TB can access the services they need, and, when they cannot, can report the problems they face in real time. This is a critical step in strengthening not only national TB responses but general healthcare responses to identify and overcome the barriers to access experienced by people affected by TB. Importantly, community volunteers should be paid and provided with the resources they need to do their work, including personal protective equipment, which has very often not been the case.

### 2.4 The need to accelerate our response in the future

Although guidance has been released on how to *maintain* TB and HIV services during the COVID‐19 pandemic [10], it is acknowledged that services considered normal pre‐pandemic must *improve* to ensure that we comprehensively meet the needs of people affected by TB. For example, the Stop TB Partnership’s “Re‐imagining TB Care” project aims to harness innovative tools and models of care to “modernize when, how and where TB care services are delivered, harness people‐centred TB innovations, and work together with high‐burden countries to provide people affected by TB with an integrated TB care model that is coordinated and comprehensive” [50].

## 3 CONCLUSIONS

Although the COVID‐19 pandemic is threatening gains made to date in the fight against TB and HIV, it also provides an opportunity to incorporate lessons learned from it to ensure we build stronger, more inclusive health systems and accelerate our efforts to advance quality TB services and realize the targets of ending TB and AIDS. This will require sufficient political will, resource allocation and joint commitment to improve and integrate the current models of care and tools, and ensure services are comprehensive and human‐rights based for all, independent of a specific disease focus.

## COMPETING INTERESTS

There are no competing interests.

## AUTHORS’ CONTRIBUTIONS

TR drafted the manuscript. All authors reviewed and contributed to the text and all authors have read and approved the final manuscript.
